# Curious afterlives: the enduring appeal of the criminal corpse

**DOI:** 10.1080/13576275.2016.1181328

**Published:** 2016-06-01

**Authors:** Sarah Tarlow

**Affiliations:** a School of Archaeology and Ancient History, University of Leicester, Leicester, UK

**Keywords:** criminal corpse, glamour, dead body, anatomy, phrenology

## Abstract

Not only did the criminal corpse have actual medicinal and magical power for Europeans, it also had social and cultural meaning as an object, a curio or secular relic. This paper considers the appeal of notorious bodies. From books bound in the skin of a criminal, to preserved and exhibited heads, from fragments of the hangman’s rope to the exhibition of the skeleton, the story of the afterlife of criminal bodies and the material culture most immediately associated with them begins with the collection and exchange of bodies and moves into contemporary preoccupations with authenticity. This paper considers the bodies of three notorious criminals of the eighteenth century: Eugene Aram, William Burke and William Corder. It ends with some reflections on the glamour of the authentic body of a notorious or celebrated individual – using the response to the discovery of the body of Richard III as an example.

## Becoming really dead

Thomas Laqueur noted recently that ‘becoming really dead- even in the West where supposedly death is a precipitous event- takes time’ (Laqueur, 2011, p. 802). This paper takes as its starting point the observation that death – even, or perhaps especially, the judicial execution of a criminal – is a process rather than a moment. Equally, biological life in the sense of a beating heart and an electrically active brain – is not always essential for the body to be a potent and meaningful locus in ongoing relationships with the living. The criminal body is powerful and dangerous, and biological death does not end that power or that danger.^1^ A current interdisciplinary project based at the University of Leicester, UK, and funded by the Wellcome Trust, is following the journey of the condemned criminal’s body from sentencing to execution and beyond. This article deals with the later stages of that process and in particular looks at those journeys that did not end in the grave. Numerous attempts to channel or harness the power of the newly executed man – such as the healing power of the hanged man’s hand, and the curative or totemic power of body parts or of objects contagiously associated with the execution (Matteoni this volume, Penfold-Mounce, 2010; Noble, 2011; Sugg, 2011) – attest to a belief that at some level the dead body of the criminal retained something of the living individual’s force and character. This paper examines the way that the criminal’s corpse was first indexical of the living man, and second that its body parts could be synecdochal of the whole criminal. Body parts were put to a variety of uses: scientific, practical, ritual and, most of all, as curios which emitted a kind of contagious glamour from the notorious criminal himself. Parts of an authentic and famous body were desirable commodities in the nineteenth century. The stories of three famous criminal bodies help to illustrate the uses to which body parts were put, and their changing post-mortem significance.

**Figure 1.**
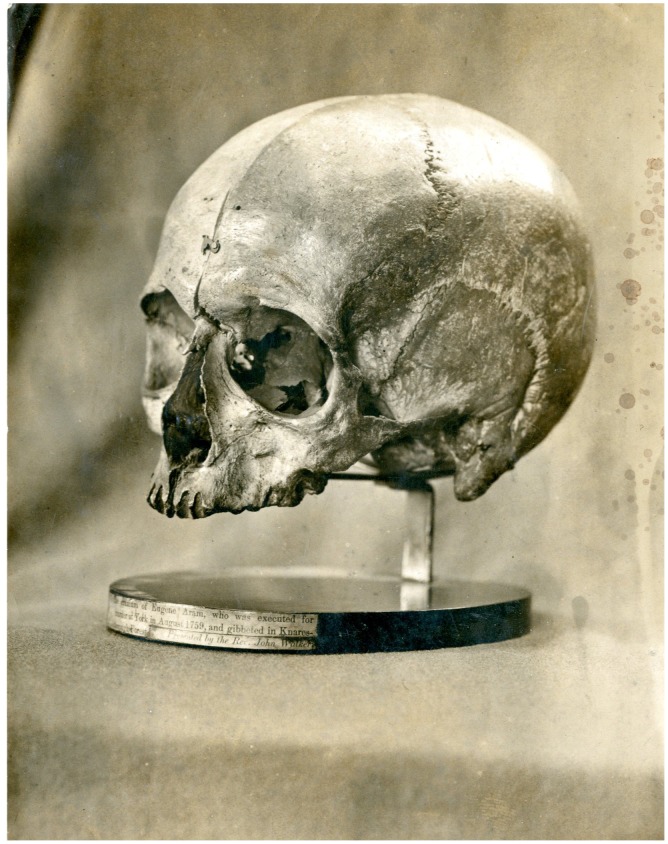
The skull of Eugene Aram. Photograph courtesy of King’s Lynn Museums.

**Figure 2.**
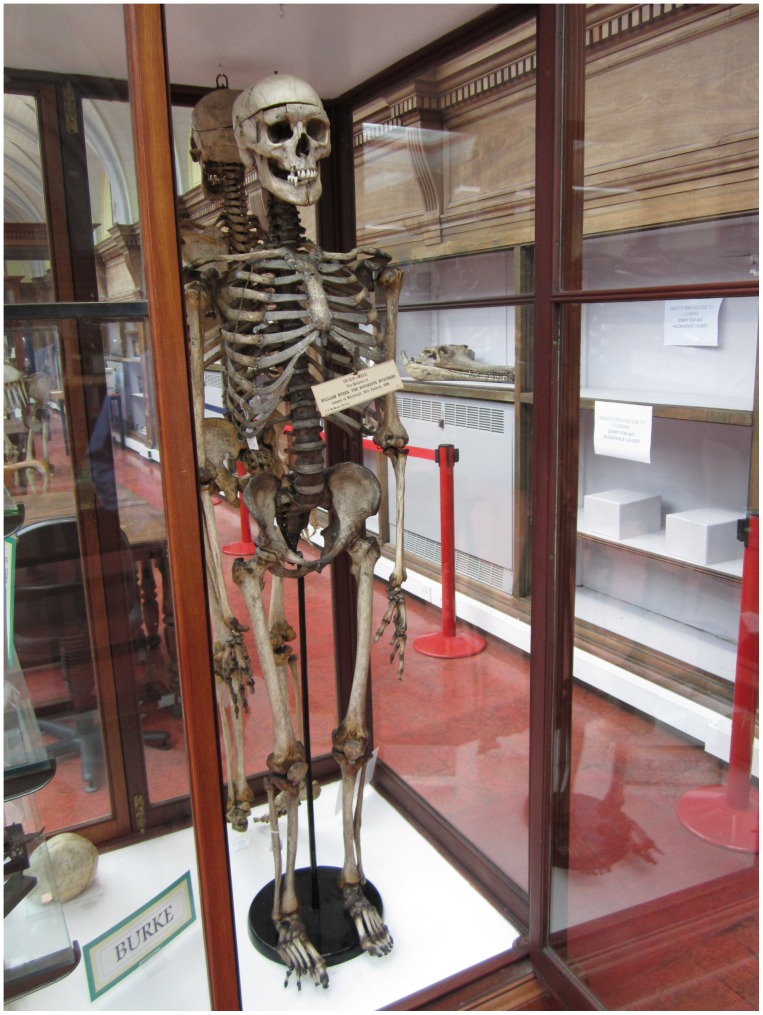
Mounted skeleton of William Burke, Anatomy Museum, University of Edinburgh (photograph by author).

**Figure 3.**
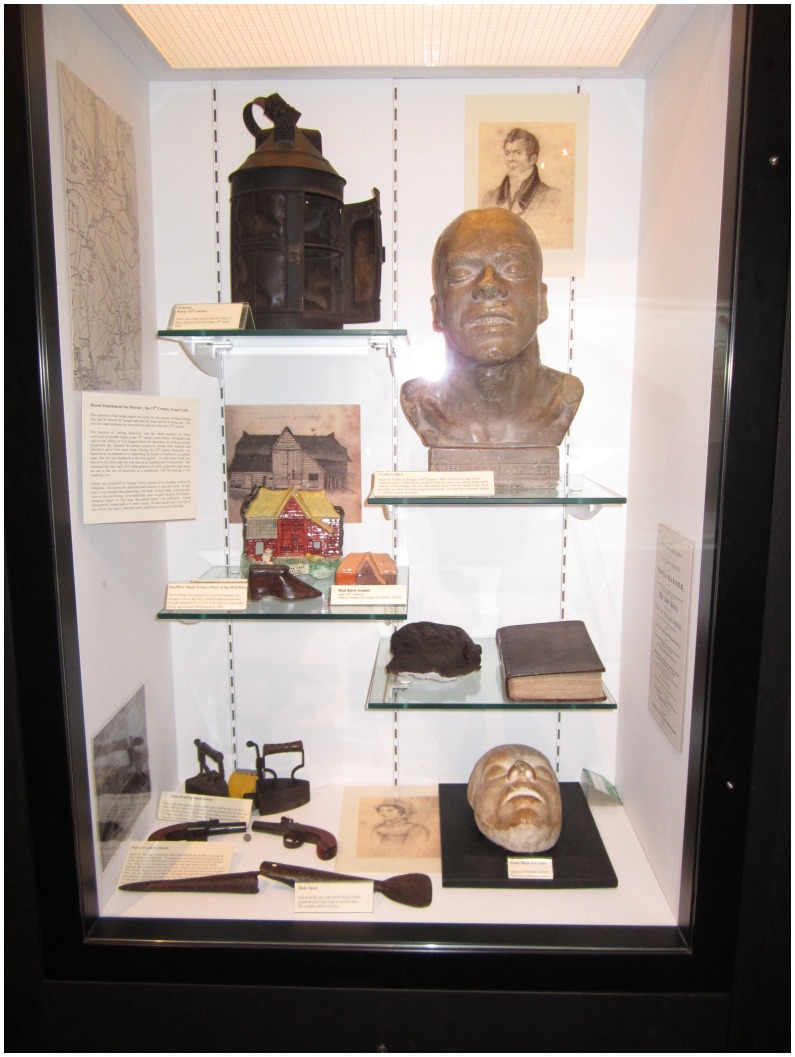
William Corder display case, Moyses Hall Museum, Bury St. Edmunds. The display includes an account of Corder’s crime and trail bound in his own skin, and a piece of his preserved skin (photograph by author).

## Case study: Eugene Aram’s head

My first case study considers the fascinating life, death and afterlife of Eugene Aram (Dobson, 1952a; Scatcherd, 1838; *The Critical Review, or, Annals of Literature,* 1759). Almost forgotten today, Aram was the focus of enormous public interest from the time of his arrest, trial and execution in 1759 up until the early twentieth century. His life, crime and death formed the subject matter of a narrative poem by Thomas Hood and a hugely popular sentimental novel by Edward Bulwer Lytton, later adapted for the stage.

Eugene Aram was born in 1704 to a family of labourers in Yorkshire in the north of England. His unusual intellectual energy and quick mind enabled him to gain an education and to discover and develop a particular gift for languages, especially ancient ones. His works on the relationships between Celtic and classical languages were serious contributions to philology. After the disappearance of his associate Daniel Clark in 1744, Aram precipitously left his wife and teaching job in Knaresborough and entered a series of other positions, eventually ending up teaching at a school in King’s Lynn, Norfolk. Thirteen years later, some bones were discovered in Knaresborough close to where Clark had last been seen. Suspicions were raised that the bones could be Clark’s and within a couple of years, following some incriminating evidence given by a mutual friend of Clark and Aram, the sheriff’s men traced Eugene Aram to his new home, arrested him and charged him with a murder committed 15 years earlier. Unwisely, Aram chose to conduct his own defence, and despite the slender evidence of the prosecution, he was found guilty and sentenced to be hanged and then gibbeted. According to criminal defence attorney Rodney Noon, it is very unlikely that any contemporary court would convict on such evidence or that such a conviction would be safe enough to withstand any appeal (Noon, 2003). However, public feeling at the time required a conviction, and accordingly one was swiftly obtained.

Because the Murder Act (1752) had come into effect between the crime and the conviction Aram was, as a convicted murderer, subject to the Act’s stipulation that his body could not be buried in consecrated ground after his execution but as a ‘further mark of infamy’ should be either dissected or gibbeted (‘hung in chains’). The latter was to be Aram’s fate and so, after his execution at York, his body was brought to Knaresborough. As was customary, his gibbet was erected close to the scene of crime, overlooking the river Nidd at Knaresborough, and his body remained there, gradually decomposing, for many years. There was no time limit on how long a body might hang in chains. Some gibbet cages remained in place for many decades until wind, weather, changing sensibilities or the exigencies of land development intervened (Tarlow & Dyndor, 2015). Aram’s gibbet seems to have remained in place for at least 25–30 years. At some point, probably before the end of the eighteenth century, a doctor called Hutchinson, then practising in Knaresborough, decided to augment his private cabinet of curiosities with the skull of Eugene Aram, and managed to remove it from its gibbet cage. Writing in 1832, the pseudonymous correspondent of a literary journal imagines Hutchinson’s attempt to extract the skull:

on a dark and stormy night, agitated by conflicting feelings, like a bridegroom on the eve of marriage, the doctor sallied forth, from the town of Knaresborough, with a ladder on his shoulder, and with the firm purpose of mounting the gibbet and detaching from the iron hoop which bound it the skull of Eugene Aram. The gibbet clung to its own property with wonderful tenacity; but the ardour of the doctor became a furore, and he succeeded in extricating another neck, almost at risk of his own.^2^ (Civis, 1832, p. 25)

Why was Hutchinson so keen to acquire Aram’s skull? It is probable that he simply wanted it as a curiosity because of its association with a significant local event – and one which had attracted national attention. However, it is evidence for the new ‘science’ of phrenology that Aram’s skull became best known. If the correspondent of the *Phrenological Journal* of 1839 is right that Norrison Scratcherd had seen the skull in Hutchinson’s possession forty years earlier (Simpson, 1839, p. 67) then it is unlikely that phrenological study was a motivation for its original acquisition, as phrenology only became popular following the publications of Gall and Spurzheimer in the early nineteenth century. Indeed, Simpson claims that Hutchinson was only ‘desirous of possessing the skull of so noted a person as Eugene Aram’ (1839, p. 67). However, within a few decades the skull was important not only as a phrenological specimen, but also as a test case, on the interpretation of which turned the credibility of phrenology as a whole.

On Hutchinson’s death the skull passed to his successor Dr Richardson. When in 1837 the young Dr James Inglis took up a post as physician at the public dispensary in neighbouring Ripon, burning with phrenological zeal, it is probable that he found out about Aram’s skull from Richardson, as a fellow medical man working in a neighbouring town (Wilson, 1973). It was Inglis who presented the skull to the Newcastle meeting of the British Association for the Advancement of Science in 1838. At that stage phrenology was not universally accepted as a science, and indeed it was always treated with suspicion and scepticism by many, or indeed most, of the British scientific establishment. Accounts of the 1838 meeting are mostly unsympathetic, like, for example, this one from the Literary Gazette:

the Doctors had a dose of phrenology foisted into their section; and hardly has that science made a more absurd appearance since Tony Lumpkin practised it upon Crackskull Common.

The skull was acquired, said Inglis in his presentation to the Society, by Hutchinson, and was inherited by his widow’s second husband, formerly Hutchinson’s apprentice, Dr Richardson, who sent it to Johann Spurzheim, one of the founders of phrenology, for examination. Spurzheim concluded it was the head of a woman: ‘this was unlucky’. Fortunately, more useful phrenological reports were soon obtained that cast doubt on Aram’s having had a violent or greedy character. Dr Inglis went on at the 1838 meeting to review the evidence on which Aram was convicted, find it inadequate, and conclude that he was wrongly convicted on the basis of both historical evidence and, most compellingly for Inglis, on the grounds that his skull, said Inglis, showed him to be a gently, scholarly man, and not a murderer: ‘we almost expected to hear a motion for the publication of [the jurors’] names, and their being branded to all posterity as persons so profoundly ignorant of phrenology as to have committed a most cruel legal murder’. (The British Association, 1838)

Aram was a celebrity criminal. Although convicted of a murder whose motivation appeared to have been purely monetary, his life and character did not fit the normal stereotype of a violent criminal. He had not lived the life of a thug but that of a scholar, a teacher and a man of apparently refined sensibilities, all of which both interested the public and occasioned later doubts about his guilt. Fictionalised retellings of his life, crime, flight from justice and eventual trial and execution were produced from the imagination of poet Thomas Hood and popular novelist Bulwer Lytton. Bulwer Lytton’s book is ludicrously melodramatic and sentimental for modern tastes but nineteenth-century English speakers around the world lapped it up. Their narrative accounts of Aram were both best-sellers; the novel was adapted for the stage and was the inspiration for a series of prints by Gustav Doré. Aram thus remained the object of popular interest well into the twentieth century. PG Wodehouse even has Bertie Wooster quoting Hood’s poem in proper Wooster style:

All I can recall of the actual poetry is the bit that goes: Tum-tum, tum-tum, tum-tumty-tum, I slew him, tum-tum tum! (PG Wodehouse, *Jeeves Takes Charge*, 1916)

Aram’s story continued to be the subject of cultural elaboration and the proliferation of narratives. But Aram’s fame was not the only kind of ‘afterlife’ he enjoyed; his actual body continued to be a thing of powerful and changing meanings long after his final breath. Aram’s skull was both a curiosity and a scientific exhibit, as we have seen, and continued its own journey through a gentleman’s private museum, through a phrenologist’s laboratory and onto the lectern of a scientific meeting. After the controversy of the 1838 meeting, the skull retired from public life for a while and did not reappear until it came into the possession of the museum of the Royal College of Surgeons in London. A letter from Hutchinson’s grandson that was sent at the time of its donation to the College in 1869 suggests that it had been passed down through the family as an heirloom (Dobson, 1952a). However, by 1869 it had become something of a strange embarrassment to its owner, an Anglican minister, who therefore sought to place it in a museum. This event co-incides with the moment of change in Victorian sensibilities around 1870 identified by Crone (2012) when overtly gory and salacious entertainments give way to more sequestered and elliptical cultural discourses of death and crime. The skull was included in the Royal College catalogues compiled by Sir William Flower in 1879 (p. 49, entry 337) and 1907. In 1993 it was given to the Old Gaol House in King’s Lynn where it remains to the present time (Figure 1).

## William Burke: the biter bit

William Burke and his accomplice William Hare were among the most notorious criminals of the nineteenth century. Burke’s name even entered the language in the verb ‘burking’, meaning to kill quietly by smothering, after their favoured method of obtaining a corpse for the surgeons without marking the body. In 1828 Burke and Hare murdered at least 16 people in Edinburgh, in order to sell their fresh bodies to the anatomist Robert Knox. Their victims were poor people, often without family and travelling away from home: people on the fringes of society who would not be much missed. When Burke and Hare were finally caught and the details of their crimes emerged, the grisly fate to which their victims were consigned both appalled and enthralled contemporary newspaper readers – not only in Edinburgh but around the world. William Hare turned King’s evidence and thus managed to avoid execution, but Burke, who was generally accounted the brighter of the two and the main instigator of the murders, bore the full vengeful wrath of society. Under the provisions of the Murder Act of 1752 Burke’s body would in any case probably be sent for anatomical dissection, but given the nature of his crime there was an especially pleasing symmetry to his post-mortem punishment. Passing sentence on Burke, the Lord Justice Clerk David Boyle decreed that not only would his body be subject to the same fate as those of his victims but also added ‘I trust, that if it is ever customary to preserve skeletons, yours will be preserved, in order that posterity may keep in remembrance of your atrocious crimes’ (Roughead, 1921).

William Burke was executed on 28 January 1829 in front of an Edinburgh crowd of up to 25,000 people, many of whom had waited for more than 24 h in the rain to secure a good view (MacGregor, 1884, p. 169–72). His body was then brought to the University for dissection, where there were riotous scenes as huge crowds surged in, hoping to see the process. Disgruntled medical students found themselves unable to get into their own teaching rooms. Eventually, after the dissection, his bones were indeed preserved, as were some other body parts, notably his skin (Figure 2).

The public outcry in the wake of the Burke and Hare case was decisive in the legislative change that culminated in the Anatomy Act of 1832 (Richardson 1989). After the passage of that act, the needs of anatomy students for fresh cadavers would be supplied by the bodies of paupers who died in the workhouse or in charitable institutions. This regulated the traffic in corpses and undermined the economic basis of grave-robbing or burking as an occupation profitable enough to outweigh the risks.

The Police Information Centre in Edinburgh holds a pocket book made from the skin of William Burke, and Surgeons’ Hall holds other items also allegedly made from Burke’s skin. The Anatomy museum in the same city still has Burke’s skeleton, on which the post-mortem cut of a craniotomy can be clearly seen, and a cast of his head. How and why did these gruesome items come to be made, traded, collected and curated? And why are they still on public display?

## William Corder: in the skin of a murderer

Moyses Hall Museum in Bury St Edmund is a typical English small town museum. It has a gallery devoted to a local artist, a few stuffed birds, some tiles and stonework from the ruins of the old Abbey. It also has, downstairs in its most popular gallery, a book bound in the skin of William Corder, the notorious Red Barn murderer. There is also a bust of Corder, a death mask, several artefacts relating to the crime and its detection, and another piece of Corder’s skin – from the side of his head and incorporating, somewhat disconcertingly, an ear. Who was William Corder, and how did parts of his body come to be preserved and exhibited in an English provincial museum?

In 1828, the year of the Burke and Hare affair in Edinburgh, another murder was thrilling newspaper readers in the south of England. William Corder’s conviction that year for murdering his fiancée Maria Marten and concealing her body gripped the nation. There are numerous inconsistencies and unusual features in the story of Corder’s conviction which have made it the subject of popular interest ever since.^3^ However the outline of the story is as follows (largely taken from Gibbs & Maltby, 1949): Farmer’s son, William Corder had been having an affair with a local girl, Maria Marten. Marten bore him a son and, despite the child’s death, pressed Corder to marry her. Finally Corder instructed the girl to meet him at the Red Barn near their homes in Polstead, Suffolk so that they could elope together. She was never seen alive again, although Corder wrote letters to her family for many months afterwards claiming that they were married and living on the Isle of Wight. Eventually Marten’s family became suspicious. Maria’s stepmother dreamed that the girl’s body was buried under the floor of the red barn, and when the barn was investigated the remains of a young woman were indeed discovered in the location that the stepmother had pointed out.

The police caught up with Corder who had by that time married somebody else and was living in London. Although many details of Corder’s apprehension and conviction are suspicious – the revelation of the location of Maria’s remains in a dream, for example, and some ambiguity about what actually killed Maria who, it was alleged, was shot, stabbed and strangled – he was quickly convicted on his own confession and sentenced to be hanged and dissected, in accordance with the Murder Act. Between 80 and 90% of all convicted murderers whose punishment came within the purview of the act were dissected rather than gibbeted, including almost all those executed after about 1810. Normally anatomical dissection, at least part of which process was open to the public, resulted in the effective annihilation of the criminal body, as the dissection of the cadaver ‘to the extremities’ was carried out (Hurren, 2012, pp. 28–29). Remains of dissected criminal corpses are rarely sufficiently substantial to leave an archaeological trace (Cherryson, Crossland, & Tarlow, 2012). But annihilation was not to be Corder’s fate.

Corder was hanged at Bury St Edmunds and his body partially opened to public view (Dobson, 1952b, p. 254). Afterwards his cadaver was used to demonstrate the principles of galvanism, as wires were attached to make his muscles twitch. Finally a dissection took place, phrenological observations on his head were made, and the remains of the body then defleshed in order to articulate and mount his skeleton. His heart was also preserved, and a considerable portion of his skin tanned, according to sources traced by Gattrell (1994, p. 256–7). The rope with which he had been hanged was auctioned and fetched up to a guinea an inch (Dobson, 1952b, p. 254). His skeleton, prepared by a Mr S. Dalton, was displayed in Suffolk General Hospital, and his skin used to bind at least one account of his trial (which survives at Moyses Hall, see Figure 3). According to a newspaper story of 1841

The skeleton of Corder, the murderer has been placed in a recess of the museum of the Suffolk Infirmary, Bury St Edmunds. It is covered with a glass case, beneath which is a box for contributions. Every visitor is expected to put silver into this box, which money is applied to the wants of necessitous patients. By an ingeniously constructed spring, the arm of the skeleton points towards the box as soon as the visitors approach it. The receipts are said to average £50 per annum. (The ingenuity seems to us to be much misapplied. There are few females who would not be terrified at such an exhibition and in some cases it might obviously produce very serious consequences). (quoted in Dobson, 1952b, p. 254)

Where parts of criminal bodies have survived dissection and anatomisation and are still around today, they are usually museum specimens – either preparations of soft tissue parts, or defleshed, mounted and articulated skeletons. These are curated in medical museums such as the Hunterian museum of the Royal College of Surgeons in London. Indeed, the skeleton of William Corder was eventually displayed there until it was cremated in 2004.

Corder’s executed body lies at the intersection of scientific and popular value. There was no obvious reason why Corder’s skeleton, rather than any other normal adult male, should be of interest to students of surgery and anatomy. However, Corder’s was not the only criminal skeleton to be exhibited at the Hunterian museum of the Royal College of Surgeons in London; when Corder’s skeleton was acquired in 1949 it was placed next to the bones of the notorious London career criminal Jonathan Wild (Dobson, 1952b, p. 254). In these cases, the line between educational demonstration and punitive display was blurred. Alberti (2011) notes that while most body parts in nineteenth-century museum collections were interchangeable with other specimens, some kept their associations with the identities of the individuals who had owned them. Possession of famous body parts enhanced the reputation of the collector and the collection. Henry Wellcome, for example, owned parts of well-known individuals including Jeremy Bentham and William Burke (Arnold & Olsen, 2003, p. 322). Alberti’s extensive explorarion of the history of collecting body parts also makes clear that the body parts themselves, as objects, could be enhanced in cultural value by the collections they were in (2011).

As with both Aram and Burke, Corder was of interest to phrenologists who hoped to be able to demonstrate the truth of their science with reference to the skull of a known villain, who had a reputation even before Marten’s murder for sly cunning and tricksy double-dealing. Corder’s bust at Moyses Hall was made by Child of Bungay as part of a phrenological reference collection and was widely reproduced for private and institutional collections around the country. Child sent a copy of the bust to Spurzheim himself, whose reply survives in the collection of Moyses Hall, and who noted, in his thorough discussion of Corder’s phrenological ‘organs’, ‘The whole of the intellectual region on the forehead is very small. The organs of individuality, tune, and language, predominate; the organs of the reflective powers are very small; the natural moral character of such a head is formed by animal feelings, deprived of self-esteem, firmness, conscientiousness, and reflection, and very little assisted by benevolence, veneration, and ideality – his internal monitor, therefore, is quite wanting’.^4^

## The curious afterlives of criminal body parts: scientific interest

The bodies of Aram, Burke, Corder and other criminals, played a real role in the development of modern medical science. Without the anatomical explorations carried out by orthodox medical, anatomical and physiological researchers, teachers and students the effective diagnostic, surgical and medical procedures that have made the modern world a safer place would have been much slower to develop. The pre-occupation of this article, however, is not the use of criminal bodies in the furtherance of mainstream medical science, which is well discussed elsewhere (e.g. Cunningham, 2010; Hurren, 2016; MacDonald, 2005; Porter, 2003; Richardson, 1987) but their powerful afterlives in less rational fields of material practice.

These three examples from the UK were all high profile cases that inspired the cultural production of art, drama, literature and ballads. However, some characteristics of their post-mortem journeys were shared widely beyond the UK by other criminals who acquired celebrity or notoriety through their crimes. In nineteenth-century America, says Michael Sappol, ‘[t]he criminological reliquary was a regular feature within the anatomical museum’ (Sappol, 2002, p. 290). As we have seen, many criminal body parts ended up in museums. Museums in the nineteenth century had a range of purposes, from the high-minded pursuit of knowledge to what was effectively making money from pornography (Sappol, 2002). These purposes were not always clearly distinct from one another. One purpose of preserving and displaying bodies is now regarded as a curiosity and a scientific cul-de-sac, but in the nineteenth century (and indeed in the early twentieth), the collection of casts of criminal heads, or even the heads themselves, served a respectable scientific purpose.

Although it was never universally accepted as an orthodox part of medicine or anatomy, phrenology represented an attempt to found a respectable science which would account for the variability of human character and behaviour by linking it to empirically verifiable characteristics of the body (van Wyhe, 2004). The science of phrenology was developed by Austrian physician Franz Gall in a series of publications during the first three decades of the nineteenth century, and widely popularised in Britain by his colleague Johann Spurzheim (Gall 1808, 1822–1825; Gall & Spurzheim, 1809, 1810, 1811; Spurzheim, 1827). It was based on the belief that the brain was formed of different ‘organs’, each of which related to a different aspect of the personality and character, so that the relative size of the various organs of the brain correlated with the propensities, sentiments and faculties of the person. By studying the shape of the head the phrenologist could determine the natural tendencies of its owner. In that way, phrenology appealed to men of science who were seeking a scientific understanding of ‘character’. Because of their selective approach to evidence and inability to show results based on good scientific method, phrenologists were not universally recognised by the wider scientific community of Britain and were eventually excluded from the British Association for the Advancement of Science. Nevertheless there were strong phrenological movements in Scotland and through much of England by the 1820s and 30s. In the case of criminals, phrenological theory required a paradigm wherein criminality inhered in the body and would be visible through the study of the head. Numerous museums continue to hold casts of the heads of murderers, originally produced for phrenological study.

It was in this world on the fringes of respectable science that Aram’s skull became a powerfully contested object. Eugene Aram’s head was discussed as a test case for phrenology, but ultimately since Aram’s own guilt and character was the object of discussion, any conclusions were circular and unverifiable. It is an interesting case for students of social history and popular belief because it rests on widely shared expectations that criminality to some extent was caused by having the body of a criminal, being a ‘criminal type’. A criminal would be recognisably such because of measurable bodily traits. All of the criminals considered in this paper were the subjects of phrenological analysis (Stone, 1829). Casts and busts of the heads of Corder and Burke were widely distributed and formed parts of nineteenth-century phrenological collections, alongside the heads of great statesmen, artists, philanthropists, writers and thinkers.

## Curious afterlives: afterlives as curios

The preservation of skulls and the making of casts and busts for the purposes of phrenology at least had the pseudo-scientific rationale that they would form part of the reference library for the developing science of phrenology. However, no comparable scientific rationale exists for preserving and using skin. William Burke’s skin not only provided the leather for a pocket book designed to hold visiting cards, but also allegedly covered a number of books, wallets and other artefacts that were exchanged or exhibited in Edinburgh in the period following his execution (Gordon, 2009, p. 177). A book covered in the skin of William Corder, and a separate piece of skin from the side of his head and including his right ear is still on display in Moyses Hall, Bury St Edmunds. That piece of skin is probably the same one exhibited in an Oxford Street shop in the mid-nineteenth century (Gatrell 1994: 258). It is alleged that the book binding was prepared by the surgeon who carried out his dissection, George Creed (Evelyn-White, 1886, p. 295).

The story of the Red Barn murder has had a remarkable afterlife, in theatre, popular literature, film and music. Corder’s actual body has had a significant role in maintaining not only the memory of his crime, but also the glamour of the criminal, to the present day. While stage, screen and cheap literature were filled with dramatic interpretations of Corder’s story (most recently a musical by David Melville was produced in California by the Independent Shakespeare Company^5^), the bones of his actual body were used to compose a bizarre and melodramatic *mise*-*en*-*scène* at Suffolk Infirmary. We see here the actual body employed in dramatic moral myth-making.^6^

The appeal of notorious body parts goes beyond scientific interest. They were collected, curated, exhibited, bought, sold, inherited, exchanged and admired. The skin, the skeleton and the head of William Corder were significant in a way that would be recognisable to an ethnographer of the Papuan highlands, or the scholar of mediaeval relics, but which challenges progressivist readings of the ‘modern’ nineteenth century. The body part had a kind of glamour because of its participation in the body of a celebrity. It was popularly attributed powers that went beyond the scientifically rational, even by those members of the scientific establishment who studied the mechanical workings of the body. The belief that, for example, Corder’s skull was ‘cursed’ and causing misfortunes to befall its owner eventually prompted its two Victorian possessors to arrange its reburial (Storey, 2004, p. 118).

## Difference and sameness

During the American Civil War soldiers on both sides collected body parts of enemy combatants from the battlefield and sent them home where they functioned as trophy relics. This practice might have been especially prevalent among Confederate soldiers, and in turn the northerners made much of the savagery and barbarism of this collection of body parts. Harrison (2010) argues that the collection of anatomical war trophies is of a piece with the efforts of nineteenth-century scientific men to build anatomical collections, and represents attempts by both sides to show that their enemy was ‘other’, and to emphasise racial difference. Harrison’s argument, however, is not wholly convincing: although there is plenty of evidence of emerging racialised discourses of difference which draw upon phrenology, there is little evidence that Confederate skull-takers were participating in those discourses. Instead, the transformation of Yankee bones into soap dishes, drinking vessels and finger rings suggests a less elevated combination of vengeance, titillation and the assertion of supremacy.

## Criminals and other glamorous bodies

The concept of glamour is a useful thought-tool in connection with criminal bodies. Glamour is a property of a person, but it can be contagious and associative also. Glamour can rub off onto objects and other bodies that have been in close contact with the source, as the blood of the decapitated criminal can carry some powerful essence into the handkerchief that is dipped into it (see Matteoni, this volume). Glamour is alluring and magical; it enhances qualities of attraction and charms those who encounter it. The term ‘glamour’ was in nineteenth century use to describe magical allure: ‘Glamour means a magic power of making an object appear to the eye different from its reality’, wrote John Carr in 1809 (p. 272).

Criminals in the eighteenth and nineteenth centuries, as today, had glamour. People have sought to connect with the glamour of crime through material objects – most notably the body itself, but if not the actual body then through material things that were in some way contagiously related to it – locations, objects owned or used by the criminal or the victim, things associated with the crime or the criminal process. The fabric of the actual Red Barn, the place of Maria Marten’s murder, for example, was extensively quarried to be reworked into toothpicks and souvenir artefacts.

We need to address the question of whether the specifically criminal nature of the celebrity corpse, in distinction to any generally famous body is especially significant. Does criminality itself impart a special or additional kind of glamour? In part the notoriety of the perpetrator of an especially shocking or gruesome crimes might add a frisson of danger to the taboo-busting titillation of seeing the body as it is not normally or decently experienced. But more pragmatically, it was the criminal body that was most easily available. ‘Normal’ celebrity bodies, such as writers, actors, artists and politicians were respectably buried and thus not available to be partitioned, preserved and exchanged for the thrill of their public. Of course, body parts could still be decently detached from their place of origin during life, such as happened frequently with locks of hair during the nineteenth century. Such hair could be kept in a locket, reworked into ‘hair art’ for jewellery to be carried close to another body, or for display in a domestic context; these works would function as synecdochal signifiers of their former owner (Hallam & Hockey, 2001, p. 4). In the nineteenth century hair art was more commonly used to remember personal and emotional attachments than to index a famous person. Nevertheless, the ubiquity in local and provincial museums of trivial objects with some association to a local celebrity bear witness to the appeal of metonymic material culture in the period. The Grace Darling museum in Bamburgh in the north-east of England celebrates the life of the daughter of a nineteenth-century lighthouse keeper who became a national heroine and sweetheart after rowing out with her father through a severe storm to rescue the survivors of a shipwreck in 1838. The museum has in its collection a teacup out of which Grace drunk when visiting family friends. Presumably this teacup, having touched her lips and thus acquired some of the glamour of her fame, was curated by the friends until it was donated to the museum.

## Authenticity, myth and the glamorous body

Authenticity is a valued characteristic of a thing or a body. We need to be reasonably certain that a body part purporting to be William Corder’s skin or Eugene Aram’s skull really *had* been part of Corder or Aram for it to have cultural value. In 2012, Glenn and Walker carried out an interesting experiment in cultural value. They bought a variety of ornaments, ash-trays, toys and similar fairings from thrift stores, none of which cost more than a couple of dollars, and gave each one to an author. Authors were then asked to write a short story featuring the object they had been given. The objects were then auctioned with their stories (although of course the stories were also freely available without the objects) Glenn and Walker then donated the proceeds to non-profit organisations dedicated to helping young or socially deprived writers. The value of the objects increased by an average of 2700% (http://significantobjects.com/about/). Objects increase massively in value (monetary value acting here as a proxy for cultural value) when there are stories that we can tell about them. Again, the authenticity of the object matters – the value of any similar plaster figurine or ash-tray would not have increased because it was just like the one about which an author told a story. The *story* need not be true, but the *object* must be the original, authentic and true subject of the fiction. In this way its glamour relates not only to the fact that it has featured in a story, but the ownership of the object creates a relationship between the purchaser and the writer. It is the material mediator of a relationship which has cultural capital and emotional weight for the possessor.

In the stories considered here – perhaps most of all in the persistent resonance of the Red Barn narrative, we are made aware of how cultural materials are used in the transformation from history to myth. The theatrical skeleton of William Corder, the anthropodermic leatherwork and other material re-imaginings of human material gave the actual weight of ‘authenticity’ to the popular myths of Corder and Marten which began to emerge in sensationalist broadsides even before Corder’s execution. From rather sordid and dysfunctional real people, the protagonists of the story were remade into a seductive and dashing villain and a wronged innocent.

Jones (2010) notes that the prevailing constructivist work on authenticity tends to neglect the significance of materiality ‘as if layers of authenticity can simply be wrapped around *any* object irrespective of its unique history and materiality’ (2010, p. 183). Instead she argues that authenticity links with particular values of modernity such as the need to classify and control, as well as a powerful need to trace and place origins. Further, she explains that the power of objects is not inherent only in the things itself, although its materiality is of central importance, but also in the networks of relationships between people, places and events that are tied into the thing. Jones relates this to the powerful and almost magical ‘aura’ of archaeological artefacts in her work. Criminal body parts are not about blood and soil in the same way that an archaeological artefact or an ancient monument could be, but her insight into the relational power of ‘authentic’ objects is very pertinent to this study. The materiality of the body part solidifies a relationship between the handler or viewer and the criminal. This relationship has some of the characteristics of intimacy – the freedom to gaze and to touch another’s body is normally reserved for the very closest and most intimate of relationships. Because the body is dead, however, the normal social constraints cannot be enforced and a simulacrum of intimacy can be had by anyone who can buy the shared material experience of the thing or the body part.

The material body constitutes a challenge to post-modern theorists of authenticity such as Holtorf (2013) who argues that the cultural quality of ‘authenticity’ as perceived by others does not depend on any quality inherent in the material thing except its capacity to plausibly simulate the characteristics of authenticity. In the case of Holtorf’s heritage studies the key characteristic is ‘pastness’; for our purposes it is ‘Burkeness’ or ‘Corderness’. Yet the latter, because they pertain to a particular body, require provenance and testing. They must have more than patina; they must be real.

## Objects and bodies

These studies all emphasise the materiality of the human body. In these cases cultural meanings are not only produced and reproduced through bodily and material practices and in material things; rather, the body itself functions as a material thing (Sofaer, 2006). The curious afterlives of criminal bodies blur the boundary between body and person – and if that boundary is a fish in a barrel to today’s cultural theorists, these curious afterlives also problematise the distinction between body and object. However, objects which were formerly part of the criminal body do retain the glamour of corporeal authenticity which gave them an extra charge. These criminal body parts were commodified in the nineteenth century in ways that made them both body and thing: partaking of an individual human, but also possessing the characteristics of a glamorous thing, to be traded, owned and displayed.

## My kingdom for a secular relic

This article concludes with a brief reflection about the most glamorous archaeological body that I have been involved with in my career so far. In 2013 some of my colleagues at the University of Leicester, against all expectations and in the unlikeliest of circumstances, discovered the remains of King Richard III under the City Council Social Services car park in Leicester city centre. Huge media interest around the world ensued, and though the university had expected a bit of a stir, the extent of popular interest and public passion took everyone by surprise. As a member of the task group charged with arranging the material details of Richard’s reburial I was amazed by the strong feelings about how and where this material body should be redeposited. The question was discussed at least three times in parliament, and the rival claims of York and Leicester to host the reburial of this controversial mediaeval king were the subject of competing petitions, campaigns by local newspapers and radio stations. I personally have also been the recipient of a series of queries from friends, acquaintances, colleagues and strangers asking if I can get them access to the bones. Gazing on, and ideally, touching, the actual bones of Richard III is a much sought-after experience. One man offered a valuable gift to the Cathedral in exchange for privileged access to the remains. None of these requests have been granted: the reburial of human remains should be neither a curiosity show, nor the subject of any financial transaction. Nevertheless, it is easy to recognise in this yearning for a material encounter the same impulses that drove the preservation, curation, exchange, display and contested ownership of past criminal bodies. Their afterlives are galvanised by our curiosity.

At the same time, the authenticity of King Richard’s bones was considered to be of overwhelming importance. A greater degree of certainty applies to the identification of the bones of Richard III than of any other archaeologically recovered identified historical figure, especially of such a great age. Identification by context of burial, pathology matching a known cause of death, carbon dating, fit with known descriptions and portraits, and genetic match with collateral descendants are all consistent with this identification (Buckley et al., 2013). Nevertheless, numerous tweets, blog postings and accostings by strangers in bars attest to widespread dissatisfaction with his identification and a demand for the kind of watertight certainty that is beyond the capacity of archaeology to supply: ‘The forensic “evidence” for these bones belonging to RIII is circumstantial. You wouldn’t convict a murderer on this type of evidence’ wrote one commentator from the blogosphere (PAB’s comment at http://howardwilliamsblog.wordpress.com/2013/09/28/what-is-truly-wrong-about-digging-up-richard-iii/). This response misunderstands the nature of archaeological evidence (it is quite right, for example, that the degree of certainty required to convict a murderer should be considerably higher than that required to identify archaeological remains, although in the cases of both Aram and Corder, it was probably slimmer). But it also illustrates a very modern desire to authenticate and to establish a secure origin. The case of Richard III shows that the glamorous attraction of authentic celebrity is as strong now as it was in the nineteenth century.

## Biographical Notes

Sarah Tarlow is professor of historical archaeology at the University of Leicester. Her research interests include archaeology of emotion and experience, the archaeology of Britain in the post-mediaeval period, and the interdisciplinary study of death and burial. She is currently leading a five-year research project on the power of the criminal corpse, generously funded by the Wellcome Trust.

## Funding

This work was supported by the Wellcome Trust [grant number WT095904AIA].

## Disclosure statement

No potential conflict of interest was reported by the author.

## Notes

1 This paper uses research gathered as part of the programme ‘Harnessing the Power of the Criminal Corpse’, funded by the Wellcome Trust. The author is indebted not only to the Trust, but also to colleagues on the project: Owen Davies, Zoe Dyndor, Elizabeth Hurren, Peter King, Francesca Matteoni, Shane McCorristine, Floris Tomasini and Richard Ward. She also thanks the University of Leicester for supporting her research, and Melissa Schrift for inviting her to participate in a panel at the American Folklore Society annual meeting 2013 in Providence, RI, for which a version of this paper was originally written.

2 The florid style adopted by Civis is a deliberate reference to Bulwer Lytton’s novel *Eugene Aram*, also published in 1832. It is, however, Bulwer Lytton’s 1830 novel *Paul Clifford* that opens: ‘It was a dark and stormy night …’.

3 It also allowed the members of the Criminal Corpse project to carry out an experiment in interdisciplinarity, in which each of us wrote an account of the Corder affair from the perspective of our own discipline. The results of this experiment include project member Shane McCorristine's monograph, William Corder and the Red Barn Murder (2014).

4 Letter from Spurzheim to Child, reproduced at http://www.stedmundsburychronicle.co.uk/rbexhib.htm.

5 Thanks to Anton Banchy for tracing this production.

6 Thanks to my colleague Elizabeth Hurren for her insights on this point.
